# Pathomorphological features of the proximal femur in crowe IV hips and their implication on stem selection during total hip arthroplasty

**DOI:** 10.1186/s12891-024-08201-7

**Published:** 2025-02-03

**Authors:** Kaveh Gharanizadeh, Elham Mohammadyahya, Mohammad Reza Bahaeddini, Shayan Amiri, Sajad Noori Gravand, Sepideh Pezeshki, Amir Aminian, Arvin Eslami, Hamed Tayyebi, Mansour Abolghasemian

**Affiliations:** 1 Bone and Joint Reconstruction Research Center, Department of Orthopedics, School of Medicine, Iran University of Medical Sciences, Tehran, Iran; 2 Department of Cardiology, School of Medicine, Mehrad Hospital, Iran University of Medical Sciences, Tehran, Iran; 3 Department of Surgery, Division of Orthopedic Surgery, University of Alberta, Alberta, Canada

**Keywords:** Total hip arthroplasty, Developmental dysplasia of the hip, High-riding hip, Femoral anatomy, Canal flair index

## Abstract

**Background:**

The best stem type and location for femoral shortening in high-riding developmental dysplasia of the hip (DDH) in not clear. We evaluated the morphology of the proximal femur on EOS™ images, focusing on the anatomical landmarks and measurements relevant to the stem selection in high-riding DDH. Our goal is to identify and define the differences in the anatomy of the proximal femur between patients with Crowe type IV DDH and normal individuals, in order to determine the appropriate neck cut location in these patients to increase the chances of successfully using a wedge femoral stem.

**Methods:**

EOS™ images of 40 hips with Crowe type-IV DDH and 40 normal hips were included. The distances between the tip of the greater trochanter and vastus ridge (GT-VR), vastus ridge and proximal border of lesser trochanter (VR-LT), greater- and lesser trochanters (GT-LT), base width of the LT, and the proportion of these distances to the femoral length were evaluated. Canal Flare Index (CFI) was also measured, at two different levels.

**Results:**

The mean GT-LT index was not different between the two groups (*p* = 0.46). The GT-VR index was smaller in the case group (*p* < 0.001), while the VR-LT index was greater (*p* < 0.001). The LT base width index was larger in the case group (*P* < 0.001). CFI was smaller at the LT level in dysplastic hips (*P* < 0.001), but the values were similar with a cut 1.5 cm above the LT (*P* = 0.67).

**Conclusion:**

In Crowe IV hips, the GT height is shorter and the LT is located far more distally along the femoral metaphysis, resulting in a narrower canal width at the upper border of the lesser trochanter. Also, the CFI at the LT level is smaller, and to fit a wedge stem, the neck cut should be made at a higher level.

## Introduction

Developmental dysplasia of the hip (DDH) is a common congenital deformity [1, 2], which if untreated, may lead to proximal migration of the femoral head over time. The altered pattern of load transfer in the proximal femur leads to distorted anatomies such as a short femoral neck and a posteriorly located greater trochanter, especially in the most severe form of DDH seen in Crowe type IV [3, 4]. Previous studies have also revealed higher neck-shaft and anteversion angles, and a more conical canal as indicated by a smaller canal flair index (CFI) [5–8].

Total hip arthroplasty (THA) is the standard treatment for neglected dislocations in adulthood. The high position of the hip and resultant soft tissue contracture are major challenges during the THA of these patients, usually mandating a femoral shortening in Crowe IV hips [9, 10]. The shortening could be performed in the proximal, sub-trochanteric, or distal portions of the femur. Controversy persists regarding the best location of femoral shortening and the best type of stem.

Although, using a low-neck cut is a common practice in arthroplasty of high-riding hips in order to minimize shortening. We have observed that after a neck cut adjacent to the upper border of lesser trochanter, the canal loses its normal wedge geometry in some Crowe IV hips, obviating the use of a wedge stem. This observation led us to hypothesize that the proximal femoral morphology of Crowe type IV hips differs from that of the normal population, not only in neck-shaft angle and anteversion angle but also in terms of the location of the greater trochanter (GT) and lesser trochanter (LT) along the femoral metaphysis.

We took advantage of the accurate measurements of EOS imaging [11–13] to evaluate this hypothesis by comparing the anatomical dimensions of proximal femur in Crowe IV- versus normal hips.

Study questions included: [1] Is proximal femoral metaphyseal flare in Crowe IV patients different from a matched normal population in terms of size and length [2] Are the relative distances between the anatomical landmarks of the proximal femur different in Crowe IV hips from normal population [3] Is CFI measured at different neck cut levels different between the two groups.

## Methods

This case-control study was approved by our institutional research ethics board. Patients with unilateral Crowe-IV hips scheduled for total hip arthroplasty at our center from March 2018 to April 2021 were included. We obtained informed consent for EOS imaging from the participants.

### Participants

We considered 61 patients with unilateral Crowe-IV hips. Exclusion criteria included presence of other congenital/developmental condition of the limb, contralateral Crowe III or IV hip, neuromuscular conditions, and history of previous fracture or surgery of the limb. 18% (11 of 61 patients) were excluded due to involvement of the opposite hip, 3% (2 patients) for neuromuscular involvement, 13% (8 patients) for previous surgery or fracture, leaving 40 patients for analysis. In the control group, 40 subjects with bilaterally normal hips who had undergone EOS imaging for other skeletal problems were matched with the cases for age, gender, and BMI. Mean age of the patients was 42 ± 14 and 43 ± 15 years for the case and control groups, respectively (*P* = 0.72), and 85% (34) were female.

### EOS imaging and measuring protocol

An EOS device was used to take biplanar radiographs. This device obtained simultaneous AP and lateral pictures (80 kV tube voltage; 200 mA tube current) with the patient in the standing position and extended knees in neutral rotation, as indicated by front-looking patellae on an AP view. To avoid overlapping of the limbs in the lateral view, the normal side was positioned slightly anteriorly. We utilized EOS imaging, which offers advantages over conventional imaging modalities. It provides higher contrast and sharpness, leading to more reliable measurements. Additionally, EOS images are captured in true magnification, eliminating magnification errors. This imaging technology allows for precise measurements even in patients with contracture, ensuring accurate assessment and analysis of the data [14, 15].

To mitigate the inaccuracies of 2-D measurements, the following parameters were measured on a SterEOS workstation on 3-D reconstructed images: The distance between GT and vastus ridge (GT-VR), VR and LT (VR-LT), and GT and LT (GT-LT), and the width of the LT base. Using three radiological landmarks: the tip of the GT as its proximal limit, the VR as the distal limit of the GT and the starting point of femoral flaring (used as a reference point because it is less affected by the altered pattern of muscle load transfer to the proximal femur), and the proximal border of the LT (the main clinical landmark for measuring the femoral neck cut during THR).

**Fig. 1:**
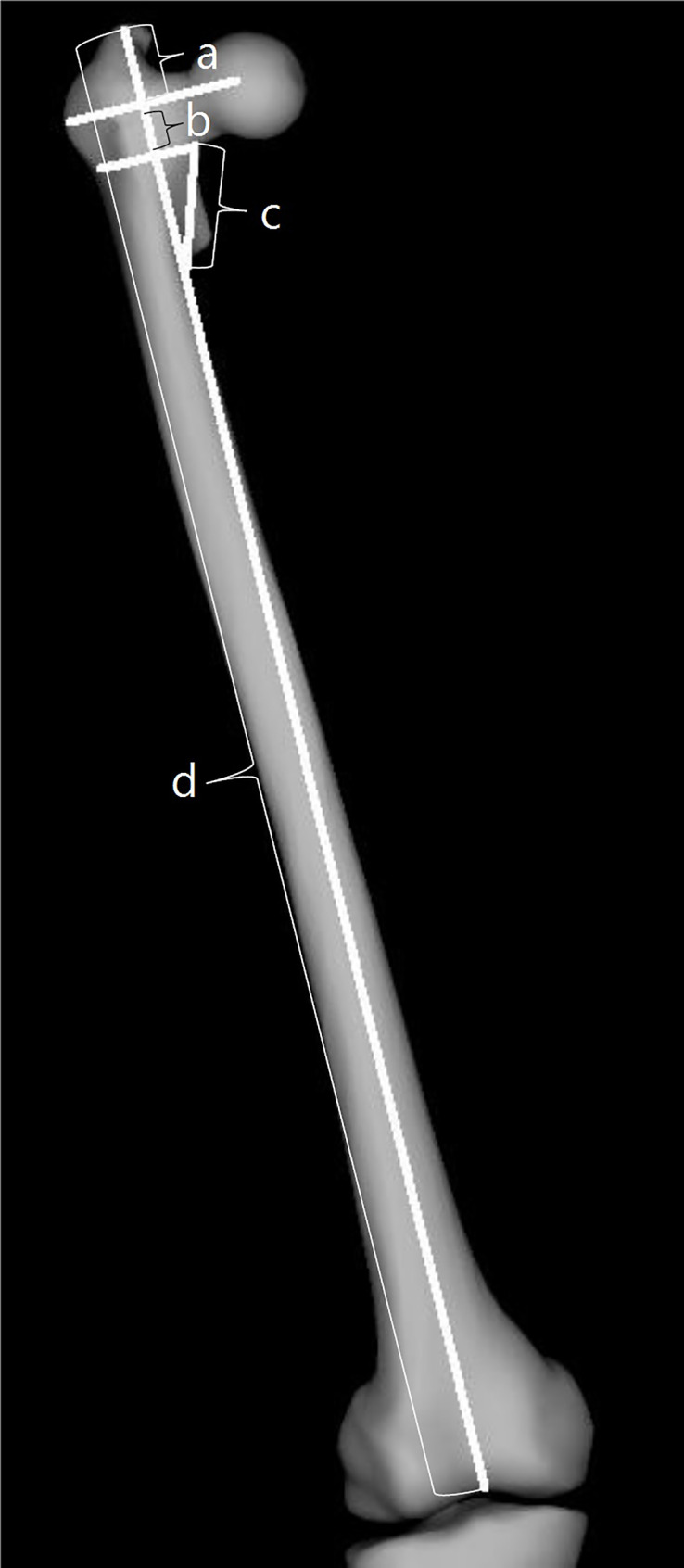
a: The distance from the tip of the greater trochanter (GT) to the vastus ridge (VR), representing the GT-VR measurement. **b**: The distance from the vastus ridge (VR) to the proximal border of the lesser trochanter (LT), representing the VR-LT measurement. **c**: The width of the LT base at the point where it aligns flush with the proximal femur. **d**: The femoral length, defined as the straight line extending from the tip of the GT to the center of the distal femoral notch

To measure the distances, a line was drawn from the tip of the GT to the center of distal femoral notch, representing femoral length. The distance between VR and proximal border of the LT was regarded as VR-LT. The distance between the tip of the GT and the VR were regarded as GT-VR, and the distance between tip of GT and proximal border of LT was as GT-LT. The distance between the points where LT becomes flash with the proximal femoral was reported as LT base-width (Fig. 1). The distances were then divided by the femoral length to eliminate the effect of patient height on the measurements, and the new values were referred to as corresponding indices.

**Fig. 2:**
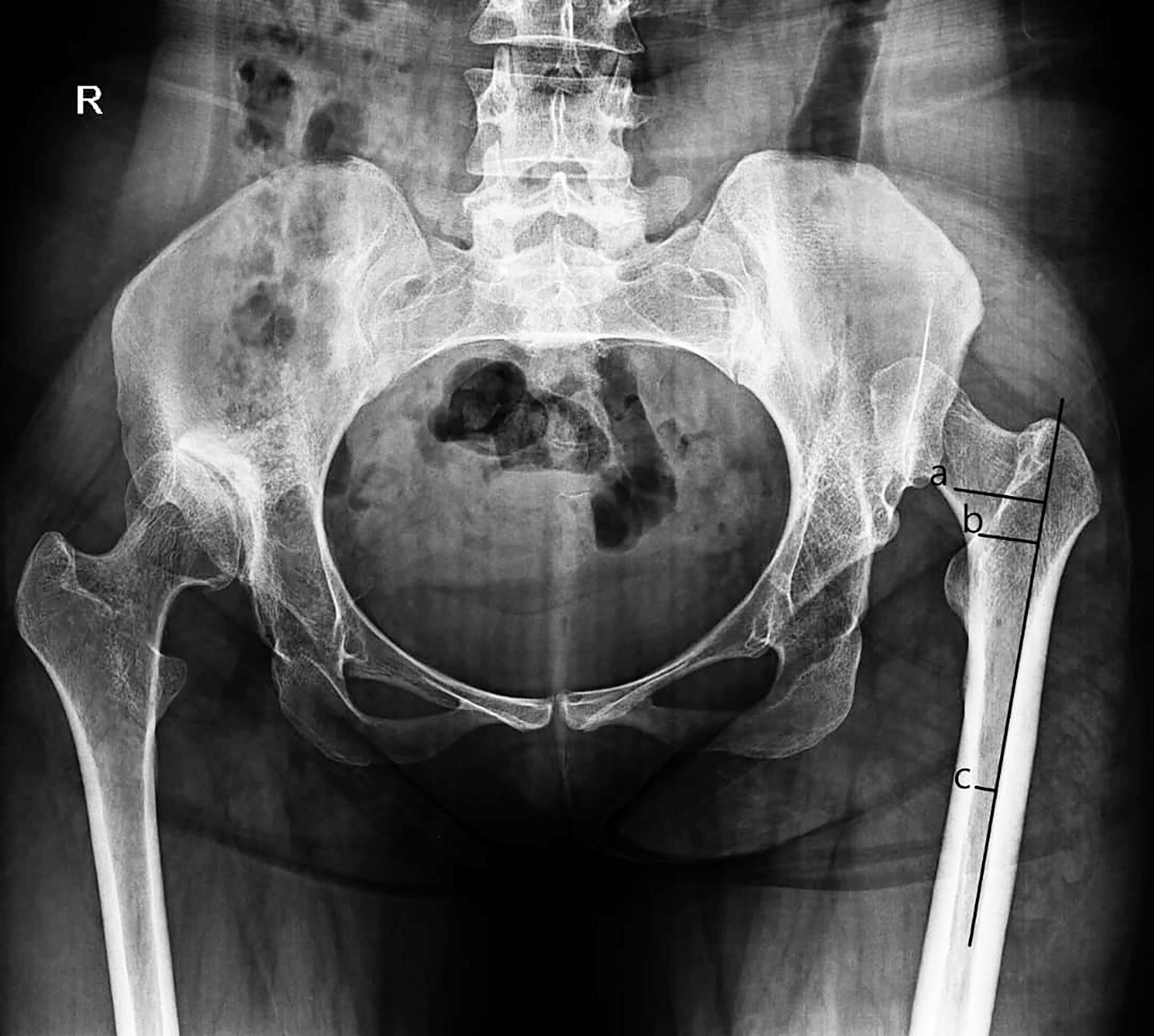
Calculation of the distance of **(a)** greater trochanter to vastus ridge and **(b)** vastus ridge to the lesser trochanter

The canal flare index was measured in 2-dimensional anteroposterior EOS images at two different levels: upper border of the LT and 1.5 cm (roughly one finger breadth) proximal to this level. Canal widths at these two levels were then divided by the canal width at the isthmus [16]. We invented a new technique for CFI measurement. Only the width of that portion of the proximal canal that could be filled by the stem was included in the measurement (Fig. 2). Then, we measured the CFI on the template of the size 0 Accolade II stem (as an example of wedge stem) and compared the findings with that of the proximal femur. All measurements were done by a fellowship-trained hip surgeon (HT) and also a fellow of hip surgery (AA). The calculated indices were compared between the two groups. For each parameter, the differences between the measurements obtained by the two observers were calculated. The mean differences were found to be less than 1 mm, demonstrating a high level of agreement between the two sets of measurements. Statistical analysis showed no significant difference between the measurements, with a p-value of 0.87, further confirming the reproducibility and accuracy of the methodology. Whenever there was a discrepancy of > 3 mm in any of the measurements, the senior author (MA) was involved.

### Statistical analysis

Statistical analysis was performed using SPSS for Windows, version 16 (SPSS Inc., Chicago, Ill., USA). The normal distribution of the data was confirmed by the Kolmogorov-Smirnov test. Categorical data were compared using Fisher’s exact test, and numerical variables were compared using independent t-test. A *P*-value smaller than 0.05 was considered significant.

## Results

Mean GT-VR index was smaller in the case group (0.06 vs. 0.08, *p* < 0.001), indicating a smaller height of the GT in Crowe IV hips. Mean VR-LT index was greater in the case group (0.06 vs. 0.04, *p* < 0.001), indicating a lower location of the LT along the femoral metaphysis. In 50% (20 out of 40) of the case group, VR-LT distance was over 20 mm, compared to 23% (9 out of 40) in the control group (*P* = 0.02). The mean GT-LT index was not different between the groups (0.12 vs. 0.11, *p* = 0.46).

The CFI was smaller at the LT level in dysplastic hips (1.8 vs. 2.4, *P* < 0.001), but the values were similar between the groups for a cut 1.5 cm above the LT (3.2 vs. 3.3, *P* = 0.67). The LT base index was larger in the dysplastic group (0.08 ± 0.02 versus 0.06 ± 0.02, *P* < 0.001) (Table 1).

**Table 1:** Comparison of proximal femur indices between normal- and high-riding hips

Type	GT-VR index	VR-LT index	GT- LT index	LT base-index	CFI at LT	CFI 1.5 cm above LT
**Normal hips (** *n* ** = 40)**	0.08 ±0.01	0.04 ± 0.01	0.11 ± 0.01	0.06 ± 0.02	2.4 ± 0.4	3.3 ± 0.3
**Crowe type IV (** *n* ** = 40)**	0.06 ± 0.02	0.06 ± 0.01	0.12 ± 0.01	0.08 ± 0.02	1.8 ± 0.2	3.2 ± 0.3
**P-Value**	< 0.001	< 0.001	0.46	< 0.001	< 0.001	0.67

GT: Greater trochanter, VR: Vastus ridge; LT: Lesser trochanter. Data are presented as mean ± SD

**Fig. 3:**
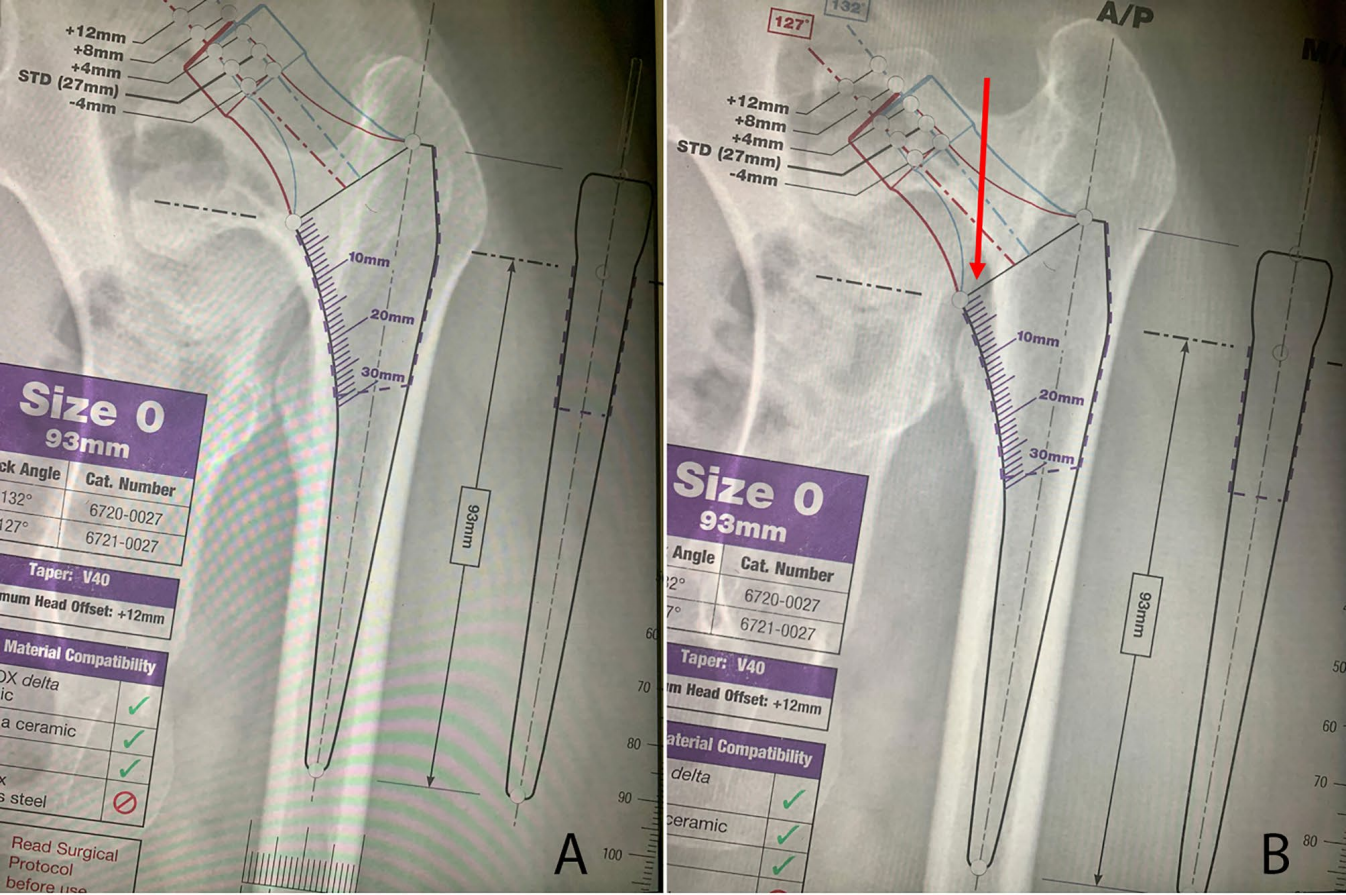
wedge stem templating over the proximal femur of a high riding DDH case with smaller CFI at the lesser trochanter (LT) level. **A**: when the neck cut made at 15 mm above LT the wedge stem template (here we used accolade II zero size template, Stryker) get a regular fit. **B**. when using low cut technique at the LT level because of the smaller CFI than the stem, it is more difficult to fit the stem regarding the mediolateral cortices. Note that red arrow shows medially the template is out of the medial cortex

The CFI of the size-0 Accolade II stem was 3.6 and 2.4 at the proximal calcar and 15 mm distal to it (compatible with the LT level), respectively, which closely corresponded to the indices of the control group (3.3 ± 0.3 and 2.4 ± 0.4, respectively). However, in high-riding hips, the CFI at the LT level was smaller than the stem CFI (1.8 vs. 2.4), resulting in a mismatch between the stem and the canal with a low cut at the LT level (Fig. 3).

## Discussion

The findings of this study suggest that the LT is positioned more distally relative to the proximal limit of femoral flaring (VR) in Crowe IV hips, where the canal’s medio-lateral diameter approaches that of the femoral diaphysis. This distal placement of the LT results in a smaller CFI at the proximal LT border in Crowe IV hips. This can lead to a pipe-shaped canal if a neck cut is made at this level. However, by making a more proximal cut around one fingerbreadth above the LT, enough CFI can be preserved to accommodate a wedge stem (Fig. 3).

In THA for high-riding DDH, the final step of reducing the prosthetic head in the cup positioned in the true acetabulum is often challenging [17, 18]. To aid in this process and protect the neurovascular elements, femoral shortening osteotomy is commonly performed. There are various techniques described, but the femur is typically osteotomized at one of three locations: the proximal femur (with a GT sliding osteotomy), the sub-trochanteric area, or the distal femur (shaft or supracondylar area) [14, 19]. Each location has its own advantages and disadvantages, and surgeons have their individual preferences. While a proximal shortening osteotomy leaves the surgeon with the only option of using a conical stem, the other two locations are usually compatible with biomechanically advantageous wedge stems.

Previous studies have observed femoral morphologic deviations in high-riding DDH [10, 17, 19, 20, 21]. For example, Liu et al. conducted a study comparing the three-dimensional morphological characteristics of the proximal femur in DDH patients and normal Chinese individuals. They found that in Crowe IV hips, the isthmus was located more proximally. They also evaluated the diameter of the medullary canal at different points in the proximal femur and noted smaller values in Crowe IV hips compared to less severe types. Their study suggested that off-the-shelf modular prostheses may not be optimal for Crowe IV hips [22]. In our study, we normalized our measurements by femoral length to minimize the potential impact of size, which we believe provides more accurate estimates for comparison purposes. Boughton et al. performed a computed tomography (CT) morphological study to demonstrate how femoral geometry changes in relation to DDH severity. They reported a decreasing length of femoral neck and increasing hip anteversion as the severity of DDH increases and concluded that these differences have implications for osteotomy and arthroplasty and these procedures should be individualized based on the observed differences [5]. No assessment of the trochanters’ locations was performed.

A novel technique was employed to measure the CFI in this study. Instead of using the conventional method, which considers the lateral cortex of the proximal femur as the lateral limit for canal width proximally, this new technique takes the line tangential to the inner cortex of the lateral cortex of the proximal shaft as the lateral limit. The reason behind this approach is that most stems do not extend lateral to this line. Consequently, the CFI measured using the conventional method has a weak correlation with stem fitness. By using this new technique, a more relevant assessment of the CFI can be obtained, leading to better evaluation of the suitability of the stem.

In dysplastic hips, there is a smaller GT altitude, while the LT is positioned more distally in relation to the proximal limit of femoral flaring (VR). In the case group, 50% had a VR-LT distance exceeding 20 mm, compared to 23% in the control group (*P* = 0.02). This difference in distances may be attributed to the tractional forces acting on the GT and LT during development. In Crowe IV hips, these forces on the GT are weakened due to the disadvantageous functional status of the abductor muscles, resulting in a shorter GT. Additionally, the force exerted by the iliopsoas muscle on the LT is oriented distally instead of its normal proximal direction. This leads to a wider base of the LT and its gradual migration distally.

**Fig. 4:**
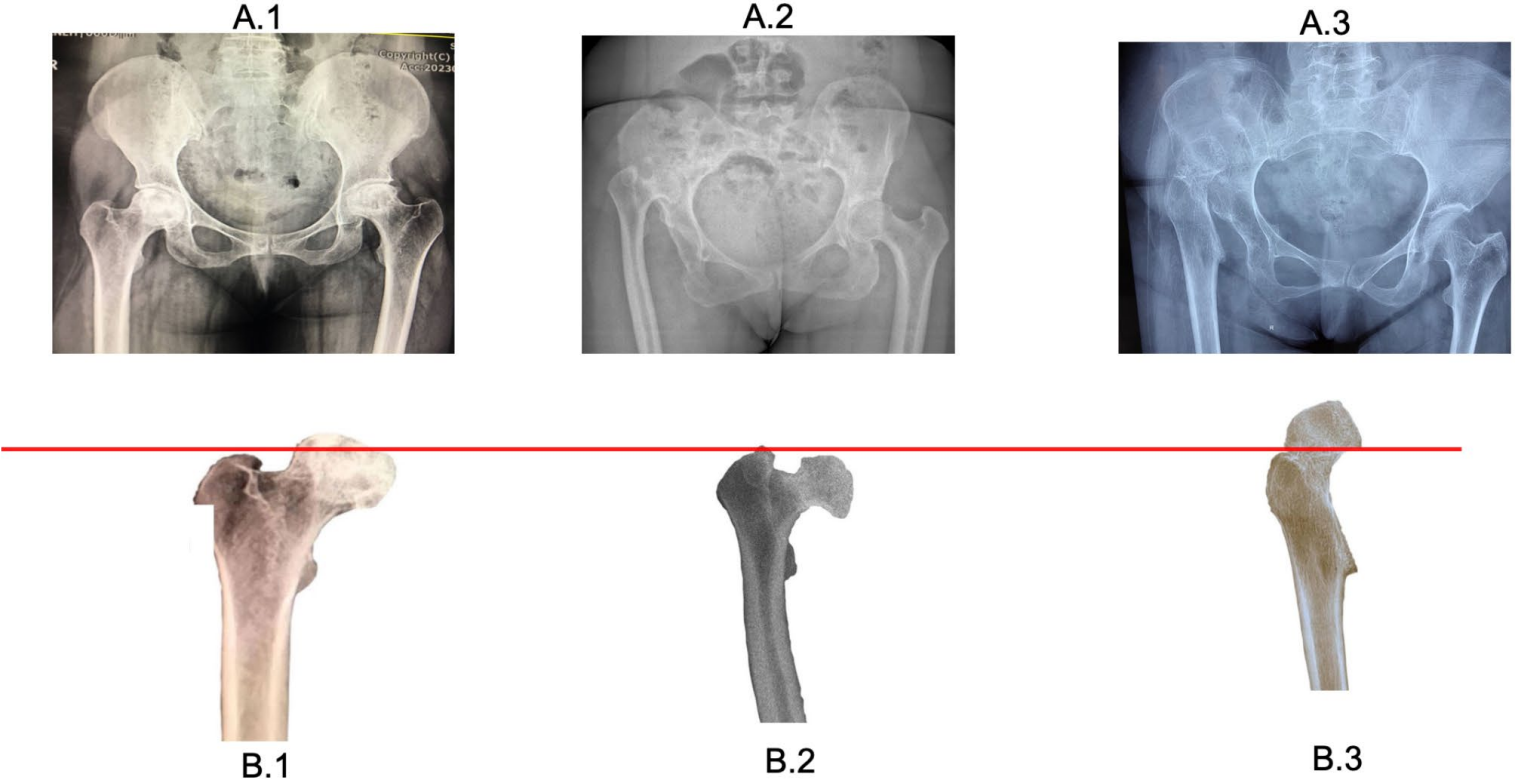
the proximal morphology of the femur between different candidates for THR with schematic drawing to show the detail pf the proximal metaphyseal flaring of the femur and the position of TH GT, VR and LT .(A1,B1) a case of femoral head osteonecrosis.(A2,B2) a high riding DDH with moderate changes including LT downward location and smaller CFI at the LT level with normal CFI at 15 mm above LT .(A3,B3) a high riding DDH with severe changes in proximal femur including very distal location of LT and almost no proximal flaring

Among high-riding cases, we identified two distinct subgroups based on the positioning of the lesser trochanter (LT) relative to the proximal limit of femoral flaring (VR). Individuals with a VR-LT distance greater than 2 cm had a relatively lower CFI at the LT level compared to those with a VR-LT distance less than 2 cm (Table 2). In Fig. 4, the first two radiographs (A1, B1) depict a typical candidate for THA due to femoral head osteonecrosis. The subsequent radiographs (A2, B2, and A3, B3) represent Crowe IV cases with VR-LT distances less than 2 cm and greater than 2 cm, respectively. As the VR-LT index increases, the feasibility of utilizing a wedge-type stem with a lower neck cut diminishes due to the loss of metaphyseal flare. In extreme cases like (A3, B3) eventually there is no real proximal flaring which mandating the use of conical stem. in contrast, wedge stems offer improved rotational and axial stability, a more physiologic load-transfer pattern, and reduced stress shielding of the calcar area compared to conical stems, which sit further away from the calcar [20, 21]. Another important point is the rotational profile of the proximal femur. When the anteversion of the femoral neck is too high, using a wedge femoral stem may lead to instability in the hip joint and result in dislocation. In some cases, excessive anteversion necessitates the use of a conical femoral stem [8].

**Table 2:** Comparison of CFI values between different subgroups of high riding DDH

Subgroups	VR-LT > 2 cm (*n* = 20)	VR-LT < 2 cm (*n* = 20)	*P*-value
**CFI at the LT level**	1.7 ± 0.5	2.0 ± 0.5	0.03
**CFI 1.5 cm above the LT**	3.0 ± 1.5	3.5 ± 1.9	0.35

CFI: Canal flare index; GT: Greater trochanter, VR: Vastus ridge; LT: Lesser trochanter. The values are presented as mean ± SD

### Limitations

The study had certain limitations. Being one of the largest series on Crowe IV hips, it still lacks sufficient statistical power for some of the analyses. The findings may also be specific to the ethnic population studied and may not be easily applicable to other populations. Furthermore, the exclusion of Crowe III hips means that the results cannot be generalized to all high-riding hips. Patients with dysplastic hip who have pseudo acetabulum, have more normal proximal femur anatomy and CFI than whose do not have pseudo acetabulum, (23) but in this study number of cases were not enough to can show this statistical difference between this groups.

Lastly, although the proposed surgical implications are supported by the authors’ clinical experiences, they still require testing in a clinical study to establish their validity.

## Conclusions

The proximal femoral morphology in Crowe type IV DDH differs from normal hips and can impact the selection of techniques during THA. One significant difference is the downward displacement of the lesser trochanter along the femoral metaphysis, resulting in a narrower canal width at the upper border of the lesser trochanter. The traditional approach of using a low neck cut at the lesser trochanter may pose challenges in accommodating a wedge stem or increase the risk of periprosthetic fractures. Therefore, a higher level of neck cut may be required based on preoperative planning. We also propose that the novel CFI measurement technique introduced in this study is more applicable and relevant to arthroplasty practice.

## Data Availability

The data that support the findings of this study are available on request from the corresponding author (H.T).
